# Extensive elemental mapping unlocks Mg/Ca ratios as climate proxy in seasonal records of Mediterranean limpets

**DOI:** 10.1038/s41598-019-39959-9

**Published:** 2019-03-06

**Authors:** N. Hausmann, A. L. Prendergast, A. Lemonis, J. Zech, P. Roberts, P. Siozos, D. Anglos

**Affiliations:** 1 Institute of Electronic Structure and Laser, Foundation for Research and Technology – Hellas, Heraklion, Greece; 2 BioArCh, Department of Archaeology, University of York, York, United Kingdom; 3 Department of Archaeology, Max Planck Institute for the Science of Human History, Jena, Germany; 4 School of Geography, University of Melbourne, Melbourne, Australia; 5 Department of Chemistry, University of Crete, Heraklion, Greece

## Abstract

Elemental analysis of biogeochemical archives is an established technique used to study climate in a range of applications, including ocean circulation, glacial/interglacial climates, and anthropogenic climate change. Data from mollusc archives are especially important because of their global abundance and sub-annual resolution. Despite this potential, they are underrepresented among palaeoclimate studies, due to enigmatic physiological influences skewing the elemental record. Understanding the patterns behind these influences will improve data interpretation and lead to the development of new climate proxies. Here, we show for the first time that extensive spatial mapping of multiple mollusc specimens using Laser Induced Breakdown Spectroscopy (LIBS) across a wider region can resolve enigmatic patterns within the elemental record caused by physiological influences. 2D elemental (Mg/Ca) maps of whole limpet shells (Patella caerulea) from across the Mediterranean revealed patterns of variability within individual mollusc records as well as within isochronous parts of specimens. By registering and quantifying these patterns, we established previously uninterpretable correlations with temperature (R^2^ > 0.8, p < 0.01). This outcome redefines the possibilities of accessing sub-annual climate proxies and presents the means to assess annual temperature ranges using oxygen isotope analysis requiring only 2 samples per shell.

## Introduction

Seasonally resolved climate proxies are a valuable tool for overcoming the biases of records reflecting only mean annual conditions^1^ as they trace the full extent of variability that is experienced throughout the year rather than only within a specific growth-window^2^. This information is essential for evaluating short-term ecological responses^3^, as well as long-term changes of seasonal variability across millenia^4^.

Most mollusc shells provide this information as biogeochemical archives of their growth-increments and are commonly analysed using oxygen isotope ratios (δ^18^Ο) or elemental ratios (e.g. Mg/Ca, Sr/Ca, Ba/Ca). Their availability along modern and ancient shorelines as well as their good preservation facilitate climatic analyses at a high temporal and spatial resolution^5,6^, in addition to ecological responses across a large population^7–9^.

Obviously sufficiently resolved geochemical studies require large sample sizes per shell specimen and with elemental analysis being more cost-effective^10^, this approach is becoming increasingly popular within the last decade^11–14^. Yet, elemental ratios in mollusc shells are more difficult to establish as climate proxies relative to δ^18^Ο values^15–17^. Studies have had mixed success in developing general equations that link elemental ratios of shell carbonate to specific sea surface temperatures (SST) even within a single species^11^. The amount of unpublished negative results is unknown but likely substantial^18^. Specifically, for *Patella* sp. shells a variety of correlations between elemental ratios and SST were found^19–22^, with no consistent explanation for any of the variabilities or anomalies that were encountered. Generally, it is argued that variations in study location as well as vital or physiological effects working between and within individual mollusc specimens prevent a comprehensive and unambiguous interpretation of their elemental records^11,21–28^.

Recently, the use of spatially extensive sampling methods (i.e. 2D-mapping) revealed trends of elemental patterning, that were able to explain some of the impact that physiological influences had in several other shell species^12,25,26^. However, these studies were limited to single specimens or parts of specimens, because of the high sophistication and cost of the employed methods (i.e. ion microprobe analysis, high-resolution secondary ion mass spectrometry) that restricted the number of samples investigated.

Here, we present the use of a rapid elemental analysis technique, Laser Induced Breakdown Spectroscopy (LIBS)^13,29^, as a means to efficiently acquire spatially extensive maps of Mg/Ca ratios within *Patella caerulea* shell specimens from across the Mediterranean. Through this, we record variations and anomalies in Mg/Ca ratios that are specific to a) research location, b) individual specimens and c) growth increments within single specimens. This information allows us to assess how much the SST record of *P*. *caerulea* shells is skewed by physiological effects and interpret the record accordingly. Even where Mg/Ca ratios did not allow absolute estimates of SST, the high-resolution maps provided information regarding the location of season of death, as well as annual minima and maxima, thus reducing the cost when subsequently sampling for oxygen isotope ratios. In all, the proposed method provides the means to access previously uninterpretable, and thus unavailable, climate proxies on a large scale.

## Results

We have identified *Patella caerulea* as an ideal species to assess elemental variations through LIBS. This species and others within the genus *Patella* have been calibrated as paleotemperature archives using δ^18^O values and have been used to assess the utility of Mg/Ca ratios as another temperature proxy^21,22^ in addition to their function as δ^18^O record^30–33^. *P*. *caerulea* is frequently found along modern shorelines^34,35^ and, similar to many other limpets, in archaeological sites^36–43^.

Elemental maps and line scans for 19 shell specimens from 9 sites were produced (Fig. 1 and Suppl. Material: Table 1, Figs S1–S13), that show the Mg/Ca intensity ratio throughout the shell record. Here we use the term ‘intensity ratio’ rather than absolute molar ratio of Mg over Ca (i.e. mmol/mol), because we compare the intensity of element specific peaks within the LIBS spectrum. These intensities are directly related to absolute concentrations, but vary depending on the selected peaks (Methods).

**Figure 1:**
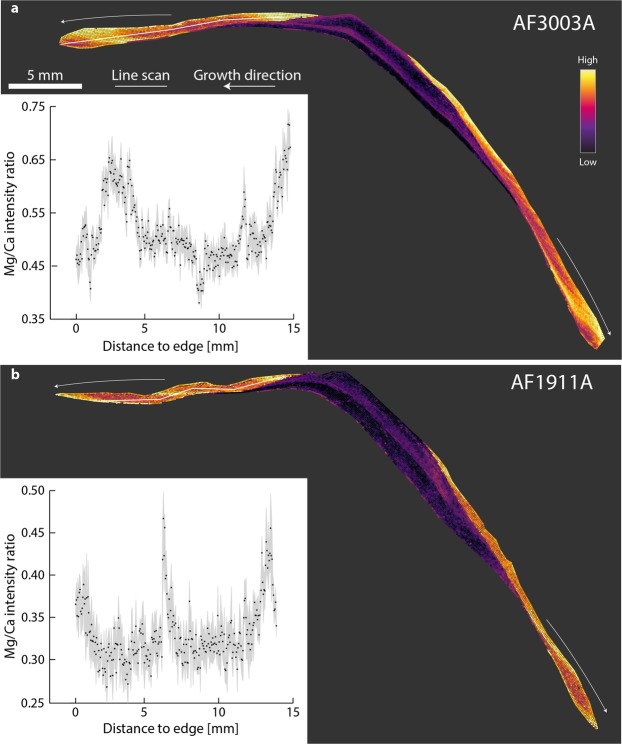
Mg/Ca maps and line-scans of whole shell sections from two specimens collected at Agia Fotini, Greece. Colours used for Mg/Ca intensity ratios range from lowest (black), to intermediate (purple) to highest (yellow) ratios found for each specimen. Line scans consist of average values from 5 spectra (black dot) and the standard deviation (grey) at each location. Collection dates for (**a**) 30 March 2018 and for (**b**) 19 November 2017. Both shells show distinct repeating patterns reflecting changes in SST (see also Suppl. Material).

In all specimens, mineralogically different layers (i.e. calcite or aragonite) gave rise to Mg/Ca intensity ratios with distinct ranges. Within calcite layers Mg/Ca intensity ratios varied between 0.15 and 0.70, while aragonite layers they were consistently below 0.10.

**Figure 2:**
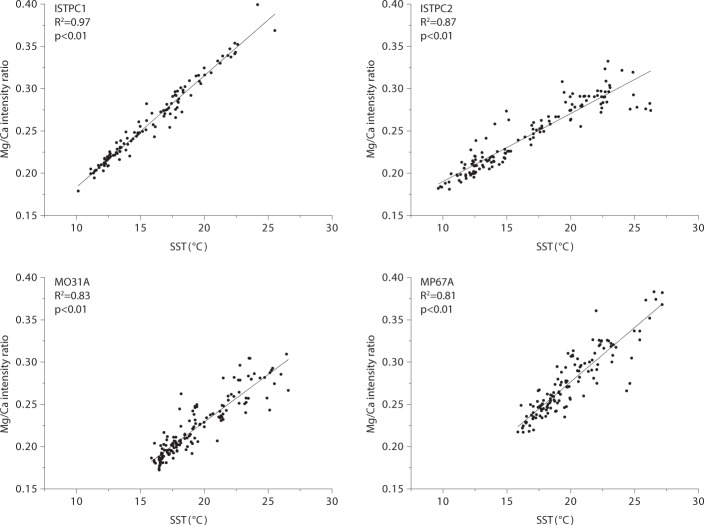
Correlation between Mg/Ca intensity ratios and SST for four specimens. (**a**,**b**) Specimens from Istria, Croatia; (**c**,**d**) specimens from Sousa, Libya. Mg/Ca ratios were taken from line scans. SST values were obtained from the KNMI climate explorer (http://climexp.knmi.nl).

Almost all specimens (90%) show in their Mg/Ca ratio maps repeating bands of high and low Mg/Ca intensity ratios that were visible throughout the entire length of growth increments and in both mineralogical layers. Only two shells (FRMPC1 and FRMPC2) both with less than one year of growth lacked these bands. The repeating patterns correspond well to seasonal changes in local SST (as derived from satellite measurements for SST: http://climexp.knmi.nl, at 1° spatial resolution) (Fig. 2, and Suppl. Material) and to previous studies of δ^18^O values within the same specimens^30^ (Suppl. Material), reaffirming a strong SST control of Mg/Ca in *P*. *caerulea*.

### Mg/Ca range variations

By including a large range of specimens, we were able to register and categorise SST related variations in Mg/Ca records as well as identify problematic specimens, which would skew the SST record if measured without being recognised.

Overall, we found that 8 out of 19 (42%) specimens showed additional patterns unrelated to SST and in 2 out of 19 (11%) specimens these additional patterns prevented reliable SST reconstruction (i.e. MP64A and MP67A) (Suppl. Material: Table S2).

These patterns were identified as a) consistent offsets of Mg/Ca intensity ratios between coeval specimens, b) short-term increases of Mg/Ca intensity ratios across growth increments but unrelated to changes in SST and c) gradual increases of Mg/Ca intensity ratios along growth-increments and towards the exterior of the shell.

**Figure 3:**
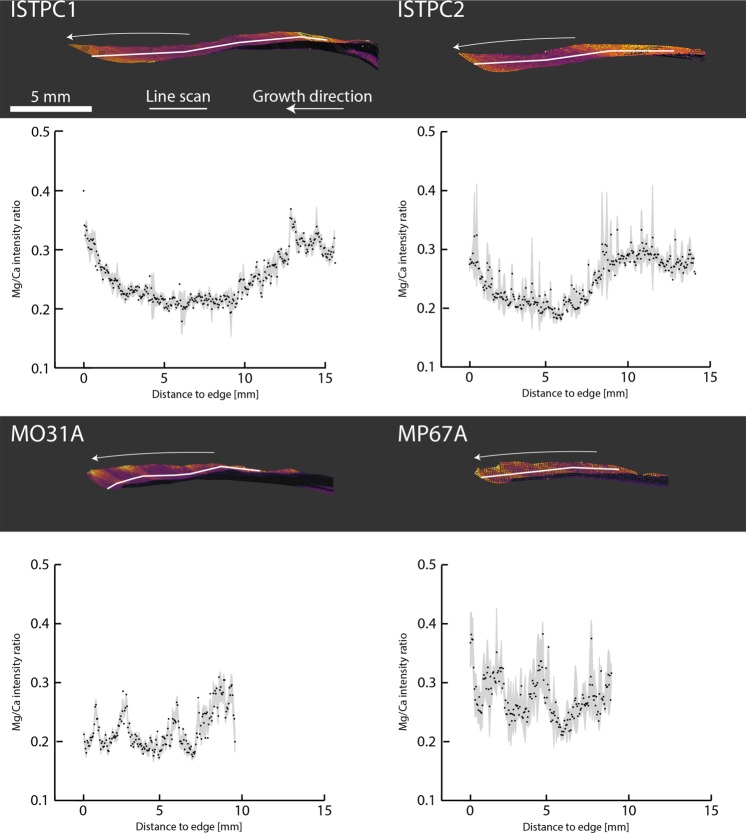
Mg/Ca maps and line-scans following the direction of growth in partial sections from the same specimens presented in Fig. 2. Colours used for Mg/Ca intensity ratio range from lowest (black), to intermediate (purple) to highest (yellow) ratios found for each specimen. Line scans consist of average values from 5 spectra (black dot) and the standard deviation (grey) at each location. (**a**,**b**) Specimens from Istria, Croatia; (**c**,**d**) specimens from Sousa, Libya.

Consistent offsets in the ranges of Mg/Ca ratios were expected to occur between study sites, because of locality-specific variations as discussed in other studies^22,44^. However, this observation was not universally true and variations among specimens coming from different localities were occasionally found to be smaller than those among specimens from the same locality. For instance, the ranges of AF1911 (0.25–0.45) and AF3003 (0.40–0.70) barely overlap, while the ranges between specimens from Istria (0.15–0.40) and Sousa (0.15–0.55) are very similar (Fig. 3).

Specimens AF1911A and AF3003A illustrate the Mg/Ca variation found between coeval specimens from the same location (Fig. 1). The molluscs experienced the same SST range, but produced different ranges for Mg/Ca intensity ratios (0.25–0.45 and 0.40–0.70, respectively). This difference can be linked partly to the LIBS specific matrix effect, as shown in other experiments^14^. The effect results from the chaotic response of irregular sample matrices (e.g. reflectivity, hardness, surface texture) under laser ablation condition in an uncontrolled environment (e.g. outside a vacuum)^45^. Additionally, the intensity of a LIBS-spectrum can change when experimental parameters vary, such as laser pulse energy or the sample distance to the focusing lens^46^. However, the differences in the Mg/Ca ranges are too high and cannot be attributed solely to a matrix effect. An influence of different habitats within the intertidal zone and differences in submersion periods can also be excluded, as both specimens were collected on the same submerged boulder.

**Figure 4:**
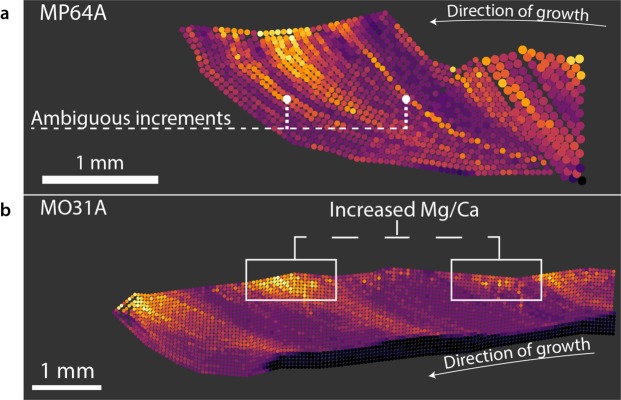
Examples of non SST related changes in Mg/Ca found within the assemblage. Colours used for Mg/Ca intensity ratio range from lowest (black), to intermediate (purple) to highest (yellow) ratios found for each specimen. (**a**) Growth increments with sharp Mg/Ca increases, that are likely not related to changes in SST. Note the increase in sampling resolution from 75 µm to 50 µm in the older increments. After the initial mapping, a higher resolution was necessary to fully interpret the anomalous increments. (**b**) Temporary increases in Mg/Ca ratios and Mg/Ca ratio heterogeneity in exterior calcite layer (M + 3).

Sharp and “short-term” increases were observed in two specimens (MP64A and MP67A) from Sousa, which seemed unrelated to SST changes as recorded in the δ^18^O values. Specifically, in MP64A elemental mapping showed annually repeating patterns, but also two short-term increases of Mg/Ca intensity ratios that were found across whole growth increments (Fig. 4a). These increases were neither mirrored in the other specimen (i.e. MO31A, Fig. 4b) collected at the same time and in the same location nor in the δ^18^Ο values of MP64A (Suppl. Material: Fig. S15b). They are thus unlikely to be related to changes in SST and instead superimpose the general sinusoidal pattern. Annual growth lines have been shown to exhibit an increase in Mg/Ca ratios due to their rich organic component^47^. Major growth lines that occur annually during the summer have also been found in a previous study of our *P*. *caerulea* specimens^30^. These lines can also occur sporadically outside of the summer season, as is the case for the ambiguous lines in MP64A. However, a major growth line was found for only one of the ambiguous lines (Suppl. Material: Fig. S15c) and we found no clear explanation that would account for both Mg/Ca ratio increases. It is possible that the increase does in fact describe a very localised temperature change. However, a more comprehensive analysis of structural and physiological factors that might also be at play, would likely provide additional explanations. Generally, these ambiguous patterns of Mg/Ca ratios are difficult to differentiate from SST related increases as they only differ in their sharper delineation or can be identified when including other coeval specimens. Furthermore, defining what constitutes a sharp and a gradual increase is more difficult in slowly growing specimens, where otherwise sufficient sampling resolutions (here 50–100 µm) are less able to capture gradual changes in the record. We found these patterns in only two of the nineteen analysed specimens, which makes them relatively rare and easily identified. Both of these specimens were collected at the same site in Sousa, suggesting that the population (or at least the collected specimens) of *P*. *caerulea* might be more prone to develop these growth increments. In fact, there might be a bias towards older specimens, as shells collected in Sousa were all between 4 and 5 years of age, while specimens from other localities were between 1 and 3 years based on the Mg/Ca ratio records.

Gradual increases in irregularity between neighbouring Mg/Ca ratios as well as increases in Mg/Ca ratios along one growth increment, and towards the exterior calcite layer of the shell (the M + 3 layer, as defined by Fenger *et al*.^48^), were found in elemental maps of 42% (8 of 19) of the analysed specimens. This trend was prominent in growth increments that have high Mg/Ca ratios, but are also apparent in low Mg/Ca areas (Fig. 4b). Due to the fact that the Mg/Ca ratio map shows no clear borders between areas with more or less noise, this spatial trend is difficult to quantify or to completely avoid when analysing the SST proxy record. Moreover, it is necessary to account for this pattern, as it can lead to offsets of Mg/Ca ratios depending on which part of the growth increment is being sampled. Ideally, future analyses of the crystal fabric will provide more detailed structural information as has been shown to be useful in studies with other mollusc species^49^. Ultimately, these offsets would change the slope and intercept of temperature equations, and potentially explain the varying temperature equations that were previously found in studies of other molluscs, such as *Tridacnidae*^44^, *Mytilidae*^50–52^, *Pinna nobilis*^53,54^, or *Pecten maximus*^55–57^.

### Re-evaluation of previous studies of Mg/Ca in Patella

Other studies that compared Mg/Ca ratios with δ^18^O values in *Patella* shells had mixed results with one study finding unreliable correlations between Mg/Ca ratios and SST^21^ and another study finding no correlation at all^22^. We can in fact use the results of the present study to facilitate interpretation of the issues found within the problematic specimens of those studies.

In the first study, Ferguson *et al*.^21^ found good correlations (R^2^ = 0.79) for an exponential temperature equation and a high potential for Mg/Ca to serve as a palaeotemperature proxy in 2 out of 4 modern specimens. One problematic specimen (*Patella caerulea* 1) provided reliable data for only the first year of its growth. In the second year the record deviates from the seasonal SST trend and Mg/Ca values were consistently higher and more erratic, although the general sinusoidal pattern remained. We can explain this deviation with the same layer-specific anomaly, which we found in our study (e.g. MO31A, and FRMPC2 in the Suppl. Material: Figure S4). We obtained increased and more variable Mg/Ca ratios in the M + 3 layers at the shell exterior, and argue that this trend is likely to have affected the data presented by Ferguson *et al*.^21^, as their sampling procedure made use of the calcite layers M + 2 as well as M + 3^48^.

In the second study, Graniero *et al*.^22^ analysed Mg/Ca ratios in two specimens of *Patella vulgata*. In both shells Mg/Ca records were insensitive to changes in SST (reconstructed through δ^18^O). In contrast to Ferguson *et al*., the *P*. *vulgata* shells were sampled only in the M + 2 layer and thus the impact of increased Mg/Ca ratios towards the exterior of the shell was expected to be smaller. However, while Ferguson *et al*. divided their milled carbonate powder samples into sub-samples for stable isotope analysis and elemental analysis, Graniero *et al*. applied two different sampling procedures for either proxy. The δ^18^O values are based on samples that were milled on a sub-monthly resolution, while Mg/Ca ratios were retrieved through laser ablation and spot sizes of 150–300 µm. This spot size is likely too large, as δ^18^O values suggest a very slow growth rate of 1.8–2.3 mm/year. We argue that it is possible that some time averaging of Mg/Ca concentrations has occurred, that obscured or skewed any potential correlations between Mg/Ca and SST. Equally, it is possible that Graniero *et al*. sampled specimens with annual or sporadic growth increments that are high in organic components^47^, as we found in two of our older specimens from Sousa.

### Combined sampling approach

While knowledge of the spatial heterogeneities of the Mg/Ca ratios and of the non SST related high Mg/Ca ratios within some specimens can help to develop an appropriate sampling strategy and to reconstruct SST more straightforwardly, caution is advised^44^. In fact, what constitutes *the* absolute Mg/Ca concentration of one increment might be difficult to define and any temperature equations using Mg/Ca might require additional geochemical information to be fully reliable.

In a recent study, García-Escárzaga *et al*.^14^ showed variable results in linear regression analysis of intensity ratios and absolute ratios of Mg/Ca measurements using LIBS and ICP-OES in the same specimens. The variation of their results is partly due to matrix effects, that skew their LIBS spectra. However, García-Escárzaga *et al*. mainly attribute Mg/Ca variability to spatial heterogeneity of elemental concentration within isochronous growth increments^14^. Variation between isochronous parts have also been found in *Leukoma thaca*^58^ and *Tridacna gigas* analysed using absolute measurements through LA-ICP-MS^25^.

Without knowledge of the Mg/Ca composition across the whole length of the measured growth increments, it is difficult to assess the patterns behind these varying regression lines. In our own assemblage, 42% of the specimens registered Mg/Ca variation of isochronous shell parts (Suppl. Material: Table S2). This complicates our ability to find a consensus between specimens or studies and makes it challenging to replicate results. Overall, many shell records might not allow researchers to derive a consistent temperature equation despite their generally good individual correlation (R^2^ > 0.80)^14,44^ (in Suppl. Material), because ranges of Mg/Ca and the resulting SST depend on the sample location within one increment.

**Figure 5:**
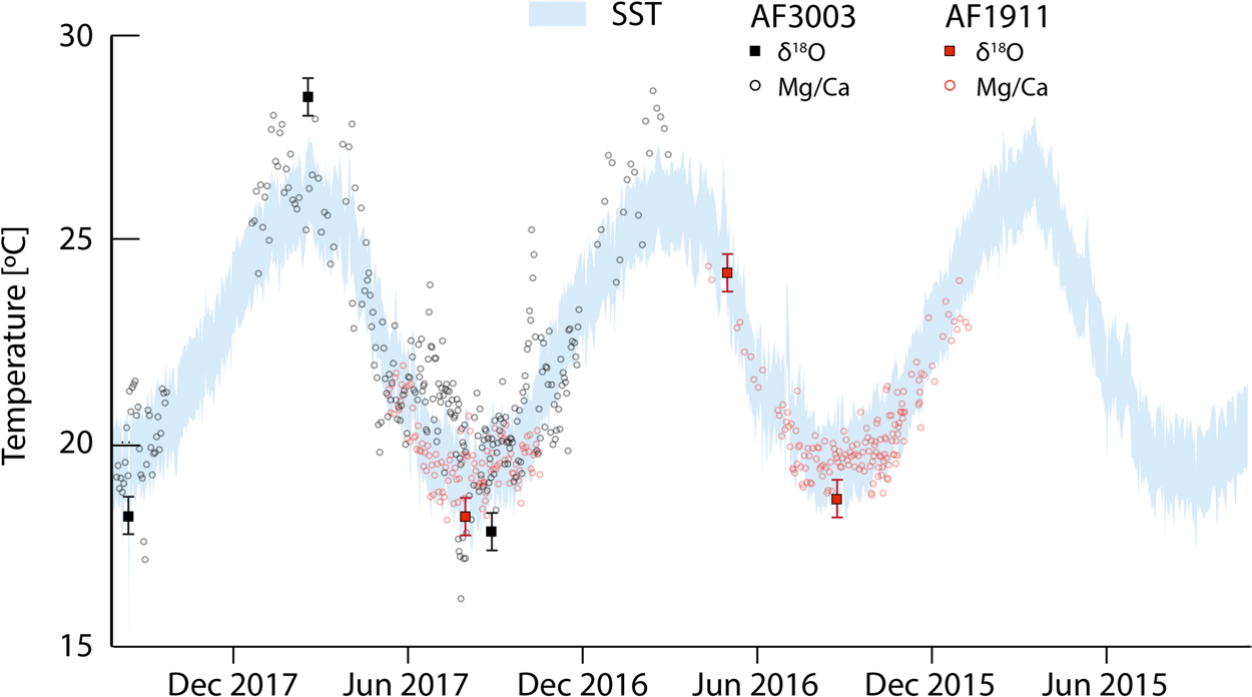
Daily SST data (blue) obtained from the KNMI climate explorer (http://climexp.knmi.nl) with SST estimates (circles) based on Mg/Ca from two shells of P. caerulea (AF3003 and AF1911). Previous line scans of Mg/Ca intensity ratios (Fig. 1) allowed us to locate annual minima and maxima and produce δ^18^O values for only these growth increments. Note the growth stop visible in the Mg/Ca record of AF1911 during summer that prevents a correct assessment of the annual SST maximum. The growth stop during autumn/winter in AF3003 is fortuitously located between temperature minima and maxima.

To retain the benefits of fast LIBS mapping (i.e. larger sample sizes and highly resolved records), and to provide comparability between specimens and accurate SST ranges, we employed an ‘anchor’ to align the temperature curves with one another. Specifically, we analysed two *P*. *caerulea* specimens (AF3003A and AF1911A) for δ^18^O values in growth increments of annual minima and maxima based on the Mg/Ca maps previously acquired (Fig. 1). Using oxygen isotope ratios, we were able to affix the Mg/Ca record to the δ^18^O derived SST range (Suppl. Material: Table S3) and thus combine the strengths of both methods (Fig. 5). This combined approach reduces δ^18^O sample numbers to only two per shell (unless multiple years are included) while retaining a high resolution overview of the record, through the elemental values in between.

### Implications and outlook

In contrast to one-dimensional sampling, our extensive LIBS mapping approach was able to confirm the general control of SST on Mg/Ca ratios in *P*. *caerulea* and, in addition, identify and quantify patterns of physiological effects on the climate record. By including multiple sites and a large set of specimens we were able to differentiate between shared and individual spatial patterns in Mg/Ca ratios that, in turn, helped us to avoid or at least acknowledge their influence on the resulting climate records. This large scale mapping strategy leads to a reorientation of the overall approach for deriving mollusc-based climate records.

The use of LIBS as an inexpensive screening method prior to sampling for comprehensive analyses (i.e. oxygen isotope analysis or clumped isotope analysis), is of consequence for future analyses of mollusc shells and potentially other sclerochronological records, as it directly addresses the main hurdle of restricted sample numbers. Our targeted approach, using only a few δ^18^O samples per shell in known locations of annual minima and maxima, allows for an increased overall amount of shells that can be analysed per study (i.e. 100–1000 specimens). This significantly increases the robustness of mollusc based climate models without reducing their subannual resolution. Specifically, this applies to studies using material from archaeological sites which contain thousands or even millions of shell specimens^59^. In fact, *Patella* bearing archaeological sites are found not only in the Mediterranean^21,39,40,42,43,60,61^ but across the world^32,62–66^ with climatic archives covering the last 160 k years^67^.

This method therefore has further implications for large-scale models comparing changes in annual temperature range through space and time, assessing the impact of climate change on whole mollusc populations rather than single specimens, or studying short-lived extreme events in past and modern contexts.

Lastly, the SST related seasonal changes in Mg/Ca ratios allow archaeologists to cost-effectively determine each specimen’s season of consumption, which is of importance for the study of past human and animal behaviour. The increased sample sizes directly translate into increased resolution within single sites and across larger site clusters, improving our general understanding of past coastal exploitation.

## Methods

### Sample Preparation

Shell samples needed little preparation as almost all (the exceptions being AF1911A and AF3003A) had previously been prepared for oxygen isotope studies^30^. As a result, only basic cleaning using ethanol and a microfibre cloth was undertaken prior to analysis using LIBS. The two samples that were collected at Agia Fotini were cleaned of their organic tissue on site, immediately after collection. Afterwards they were sectioned in half along their main growth axis (Suppl. Figure S14) using a Buehler Isomet low-speed saw and subsequently polished using metallographic grit paper (P800, P1200).

### Laser Induced Breakdown Spectroscopy (LIBS)

To acquire spatial information about the Mg/Ca composition of the shell sections, we employed a customised LIBS microspectrometer which in combination with a computer controlled sample stage enables scanning of shell sections in two modes: line-scan or 2D-mapping. The LIBS system makes use of an infrared (1064 nm) Nd:YAG laser (Spectron Laser Systems), that emits pulses of 10 ns duration, which were focused directly onto the sample surface using an objective lense (10x magnification, 28 mm focal length, LMH-10× −1064, Thorlabs) with infrared anti-reflection coating. Each pulse has an energy of ~10 mJ and irradiates the sample on the exposed surface in an area of 50 µm diameter (Suppl. Figure S16). Within this area, the laser irradiation creates a luminous plume of ionised sample material. A quartz fibre collects the light emitted by the plume and guides it into a CzernyTurner spectrograph (Jobin Yvon, TRIAX 320). We used a 600 l/mm grating on the spectrograph and an ICCD (intensified charge coupled device) detector (DH520–18F, Andor Technology) to measure the spectrum using a delay of 0.5 microseconds and a gating of 1 microsecond. The ICCD is gated by means of a digital delay pulse generator (DG535, Stanford Research Systems) and synchronized to the Q-switch of the laser. All measurements were performed at ambient temperature and pressure. The individual components were controlled through a dedicated software developed using LabView 2012 (National Instruments).

Using this software, via a CCD camera (FLIR Grasshopper 3), we defined the outline and tilt of the shell section that was to be mapped. The shell outline was then automatically filled with sample location coordinates at a given resolution for interpolation (50–100 µm). For each sample location we chose to accumulate 5 spectra and used the resulting accumulated spectrum to measure the intensity at specific peaks (i.e. Mg II 279.553 nm and Ca II 315.887 nm). The resulting intensity ratio was thus averaged over 5 measurements. Prior to each measurement, we also used 2 laser pulses to clean the area that was to be irradiated and to expose a new sample surface, to prevent any surface debris from affecting the measurement. Sampling speed was ~0.9 s per location and each shell map consisted of 6,000 to 8,000 individual locations. This translates to 90–120 minutes per specimen. Where applicable (e.g. sample MP64A), we resampled specimens at an increased resolution of 50 µm for a more detailed elemental map (Fig. 5a).

All shell section surfaces were mapped in their entirety, however, because the majority of shell specimens (excluding AF3003 and AF1911) had been milled or drilled prior to this study, only one side (either anterior or posterior slope) of the shell section was preserved for mapping.

Line scans from the growth edge towards the apex of the shell were recorded after each map at a resolution of 50 µm and 5 spectra per location. Line scans were uninterrupted and followed along a central path that stayed clear of aragonite layers. Where the shell thickness allowed it, line scans were focused on a distance of 1–2 mm to the exterior border of the shell, to minimise the influence of physiological effects in the M + 3 layer. Each line scan comprised of 200–400 sampling locations and was again sampled with~0.9 s per location. This translates to 3–6 minutes per specimen.

### Oxygen Isotope Measurements

Further to existing δ^18^O values from high-resolution carbonate samples published by Prendergast and Schöne^30^, additional δ^18^O data was collected from the previously mapped shell sections of AF1911A and AF3003A at selected growth increments of annual minima and maxima as indicated by the LIBS maps. Carbonate samples were taken using a New Wave Research MicroMill and a 80 µm diamond-coated drill. 150 μg of shell powder was weighed out into 12 ml borosilicate glass vials and sealed with rubber septa. Following reaction with 100% phosphoric acid, gases evolved from the samples were analyzed to stable carbon and oxygen isotopic composition using a Thermo Gas Bench 2 connected to a Thermo Delta V Advantage Mass Spectrometer at the Department of Archaeology, Max Planck Institute for the Science of Human History. Oxygen isotope (δ^18^O) values were calibrated against international standards (IAEA NBS 18, IAEA 603, IAEA CO8) registered by the International Atomic Energy Agency. Replicate analysis of an in-house calcium carbonate standard (MERCK CaCO_3_) suggests that machine measurement error is *c*. ± 0.02‰ for δ^18^O. The δ^18^O_shell_ values were used to calculate sea surface temperature (SST) using the equation of O’Neil *et al*.^68^ after applying a negative offset of 0.72‰^30^:

$${\rm{SST}}(\mathop{^\circ }\limits_{\_}C)=16.9-4.38\,\ast \,({{\rm{\delta }}}^{18}{{\rm{O}}}_{{\rm{Shell}}}-{{\rm{\delta }}}^{18}{{\rm{O}}}_{{\rm{Water}}})+0.1\,\ast \,{({{\rm{\delta }}}^{18}{{\rm{O}}}_{{\rm{Shell}}}-{{\rm{\delta }}}^{18}{{\rm{O}}}_{{\rm{Water}}})}^{2}$$

A mean annual δ^18^O_water_ value of 1.68‰ was used in the SST calculations, which we derived from sea surface salinity (SSS) data according to the following equation by Pierre^69^:

$${{\rm{\delta }}}^{18}{{\rm{O}}}_{{\rm{Water}}}=(0.25\ast {\rm{SSS}})-8.2$$

Monthly mean SSS and daily SST data were collected for each shell collection site from the KNMI Climate explorer website (http://climexp.knmi.nl).

### Construction of linear correlation graphs using Mg/Ca with δ^18^O and SST

For most shell specimens (excluding AF3003 and AF1911) both geochemical datasets (Mg/Ca and δ^18^O) were acquired from different parts of the shell (i.e. anterior and posterior slope). These parts likely experienced different growth rates^64^, and thus different degrees of time averaging, and because the sampling procedure for the study of oxygen isotopes made use of a varying sampling resolution (30–100 µm) rather than consistent sampling (50 µm), it was necessary to align both records computationally, using Dynamic Time Warping^70^. This method preserves the sequence of data points but warps their individual distances from each other to achieve best alignment of both records. We followed the same procedure for the alignment of Mg/Ca ratios and the instrumental SST records.

## Supplementary information

Dataset 1
