# Supplementary material for: Extensive elemental mapping unlocks Mg/Ca ratios as climate proxy in seasonal records of Mediterranean limpets
Source: Sci Rep. 2019 Mar 6;9:3698. doi: 10.1038/s41598-019-39959-9 (PMC6403426; doi:10.1038/s41598-019-39959-9)
Supplement: Supplementary file 1 — Dataset 1 [file 41598_2019_39959_MOESM1_ESM.docx]

Supplementary Information

**Extensive elemental mapping unlocks Mg/Ca ratios as climate proxy in seasonal records of Mediterranean limpets**

*Hausmann, N.*^1,2,3^ , Prendergast, A.L.^4^, Lemonis, A.^5^, Zech, J. ^3^, Roberts, P. ^3^, Siozos, P.^1^, Anglos D.^1,5^*

^1^Institute of Electronic Structure and Laser, Foundation for Research and Technology – Hellas, Heraklion, Greece

^2^BioArCh, Department of Archaeology, University of York, York, United Kingdom

^3^Max Planck Institute for the Science of Human History, Jena, Germany

^4^School of Geography, University of Melbourne, Melbourne, Australia

^5^Department of Chemistry, University of Crete, Heraklion, Greece

*niklas@palaeo.eu

Supplementary Table S1, Origin and collection dates for analysed shell specimens

| Country | | Latitude | Longitude | Shell ID | Date of collection |
| --- | --- | --- | --- | --- | --- |
| Croatia | Istria | 44.875579 | 13.846959 | ISTPC1 | 30/07/80 |
|  |  |  |  | ISTPC2 | 30/07/80 |
| Greece | Agia Fotini | 35.144391 | 24.514549 | AF1911A | 19/11/17 |
|  |  |  |  | AF3003A | 30/03/18 |
| Israel | Akko | 32.920317 | 35.072141 | AKKPC2 | 10/03/02 |
|  |  |  |  | AKKPC3 | 10/03/02 |
|  | Tel-Aviv | 32.068793 | 34.761464 | FRMPC1 | 27/07/14 |
|  |  |  |  | FRMPC2 | 27/07/14 |
| Libya | Sousa | 32.907994 | 22.044392 | MO31A | 15/04/12 |
|  |  |  |  | MP64A | 15/04/12 |
|  |  |  |  | MP67A | 15/08/12 |
|  |  |  |  | MP68A | 15/09/12 |
| Malta | Bahar-ic Caghaq | 35.949647 | 14.428678 | MA10 | 15/08/10 |
| Tunisia | Djerba | 33.870888 | 10.962373 | TUNPC1 | 30/04/85 |
|  |  |  |  | TUNPC2 | 30/04/85 |
| Turkey | Antalya | 36.883919 | 30.679270 | ANTPC1 | 01/09/00 |
|  |  |  |  | ANTPC2 | 01/09/00 |
|  | Kizkalesi | 36.457748 | 34.143991 | KIZPC1 | 30/06/13 |
|  |  |  |  | KIZPC2 | 30/06/13 |

Supplementary Table S2, Occurrence of anomalies within specimens.

| Sample | Site | Ambiguous increments | M+3 bias |
| --- | --- | --- | --- |
| ISTPC1 | Istria |  |  |
| ISTPC2 |  |  |  |
| AF3003 | Agia Fotini |  | consistent |
| AF1911 |  |  |  |
| AKKPC2 | Akko |  |  |
| AKKPC3 |  |  | inconsistent |
| FRMPC1 | Tel Aviv |  |  |
| FRMPC2 |  |  | inconsistent |
| MO31A | Sousa |  | inconsistent |
| MP64A |  | Yes | inconsistent |
| MP67A |  | Yes | inconsistent |
| MP68A |  |  |  |
| MA10 | Bahar-ic Caghaq |  |  |
| TUNPC1 | Djerba |  |  |
| TUNPC2 |  |  |  |
| ANTPC1 | Antalya |  | consistent |
| ANTPC2 |  |  | consistent |
| KIZPC1 | Kizkalesi |  |  |
| KIZPC2 |  |  |  |
| **n=19** | **n=9** | **11%** | **42%** |

Supplementary Table S3, Oxygen isotope values from annual minima and maxima within AF3003 and AF1911 specimens.

| Sample | δ^18^O | ± | SST | ± |
| --- | --- | --- | --- | --- |
| AF3003 | **2.1** | 0.2 | **18.2** | 0.9 |
|  | **-0.2** | 0.15 | **28.8** | 0.7 |
|  | **2.2** | 0.1 | **17.8** | 0.4 |
| AF1911 | **2.1** | 0.2 | **18.2** | 0.9 |
|  | **0.7** | 0.1 | **24.2** | 0.4 |
|  | **2.0** | 0.1 | **18.6** | 0.4 |

## Elemental maps and line scans for all shell specimens not included in the main text.


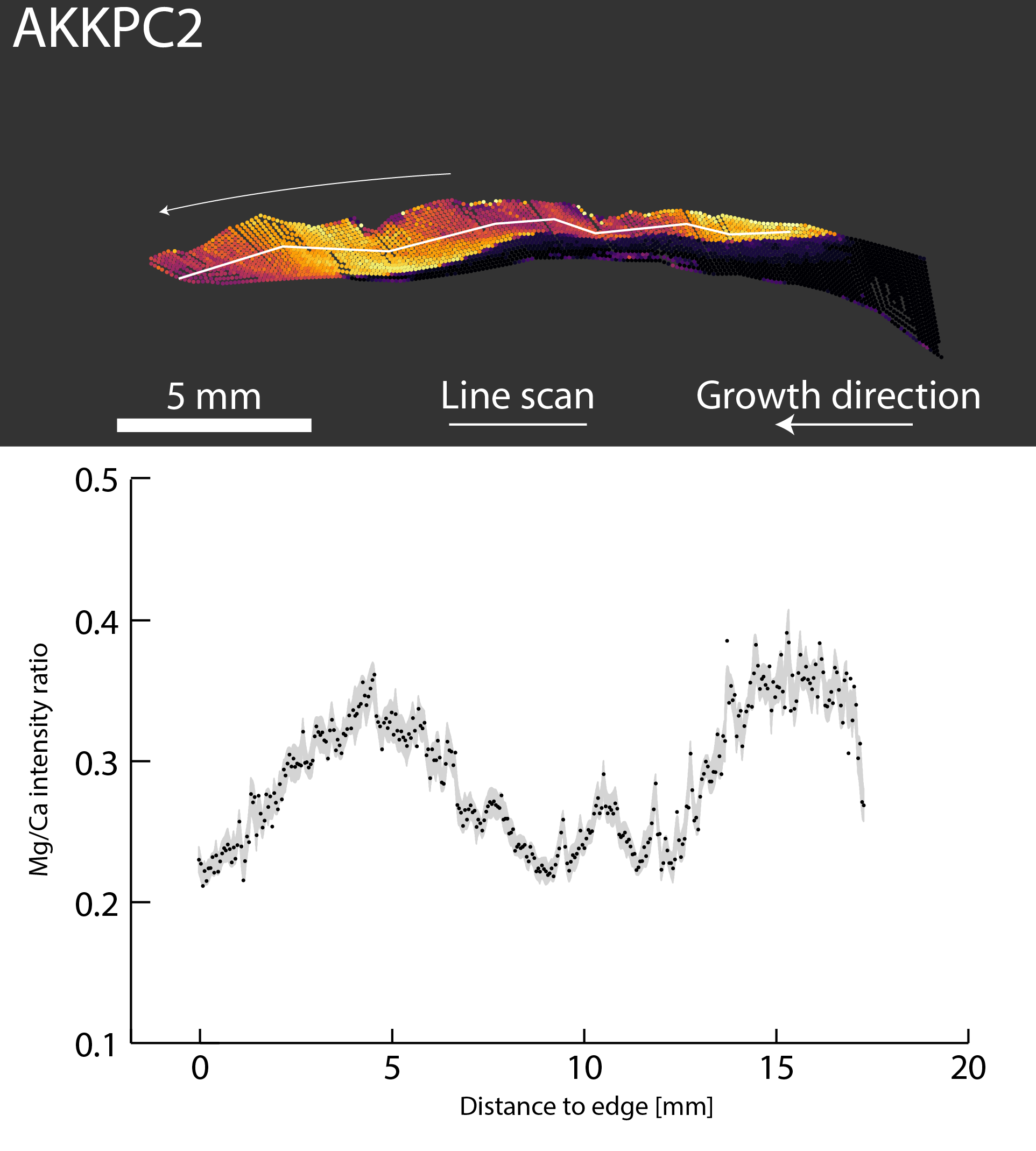


Supplementary Fig S1, Mg/Ca maps and line-scans following the direction of growth in partial sections of AKKPC3. Colours used for Mg/Ca intensity ratio range from lowest (black), to intermediate (purple) to highest (yellow) ratios found for each specimen. Line scans consist of average values from 5 spectra (black dot) and the standard deviation (grey) at each location. The line scan follows the centre of the M+2 layer from the growth edge towards the apex.


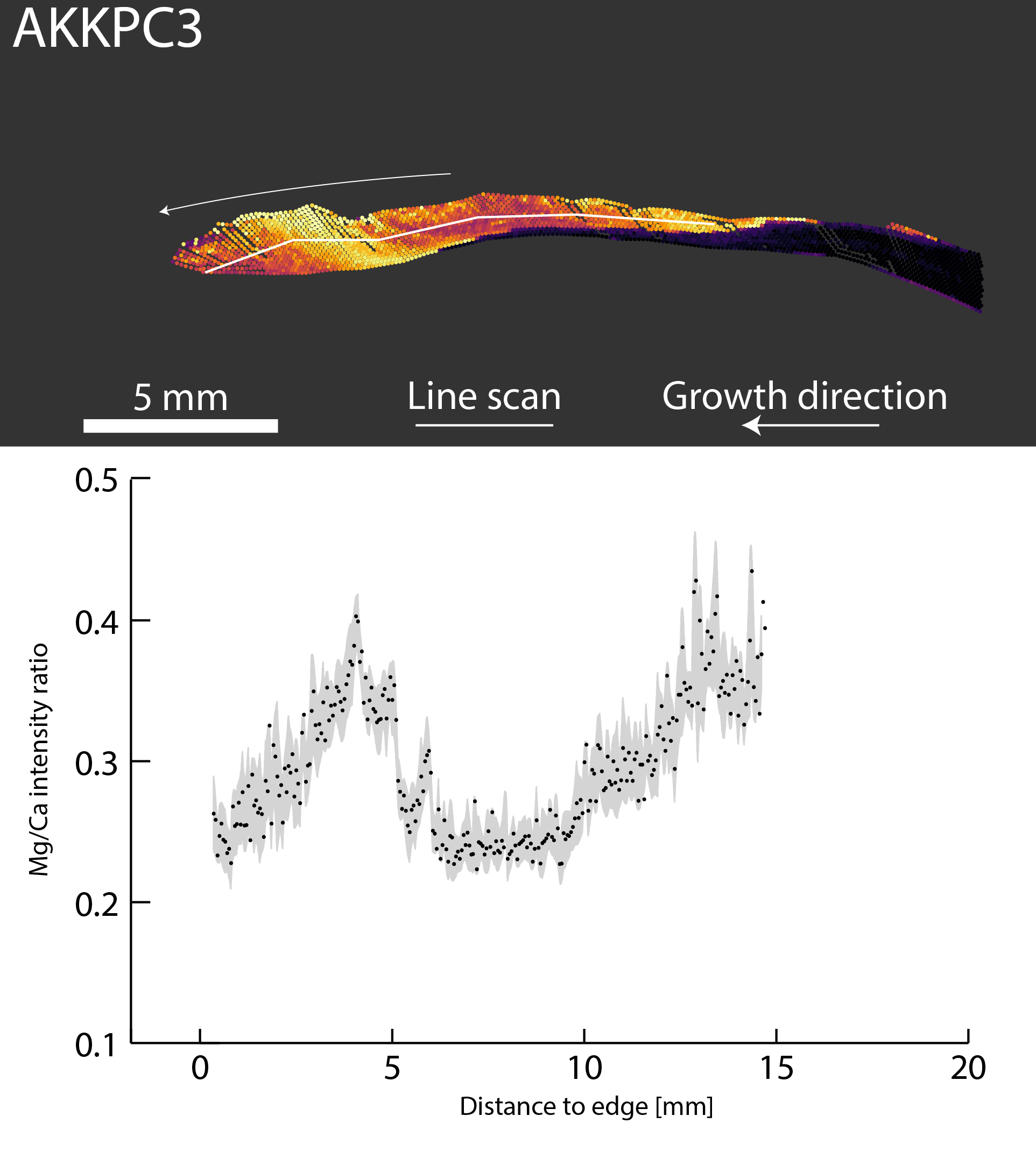


Supplementary Fig S2, Mg/Ca maps and line-scans following the direction of growth in partial sections of AKKPC3. Colours used for Mg/Ca intensity ratio range from lowest (black), to intermediate (purple) to highest (yellow) ratios found for each specimen. Line scans consist of average values from 5 spectra (black dot) and the standard deviation (grey) at each location. The line scan follows the centre of the M+2 layer from the growth edge towards the apex.


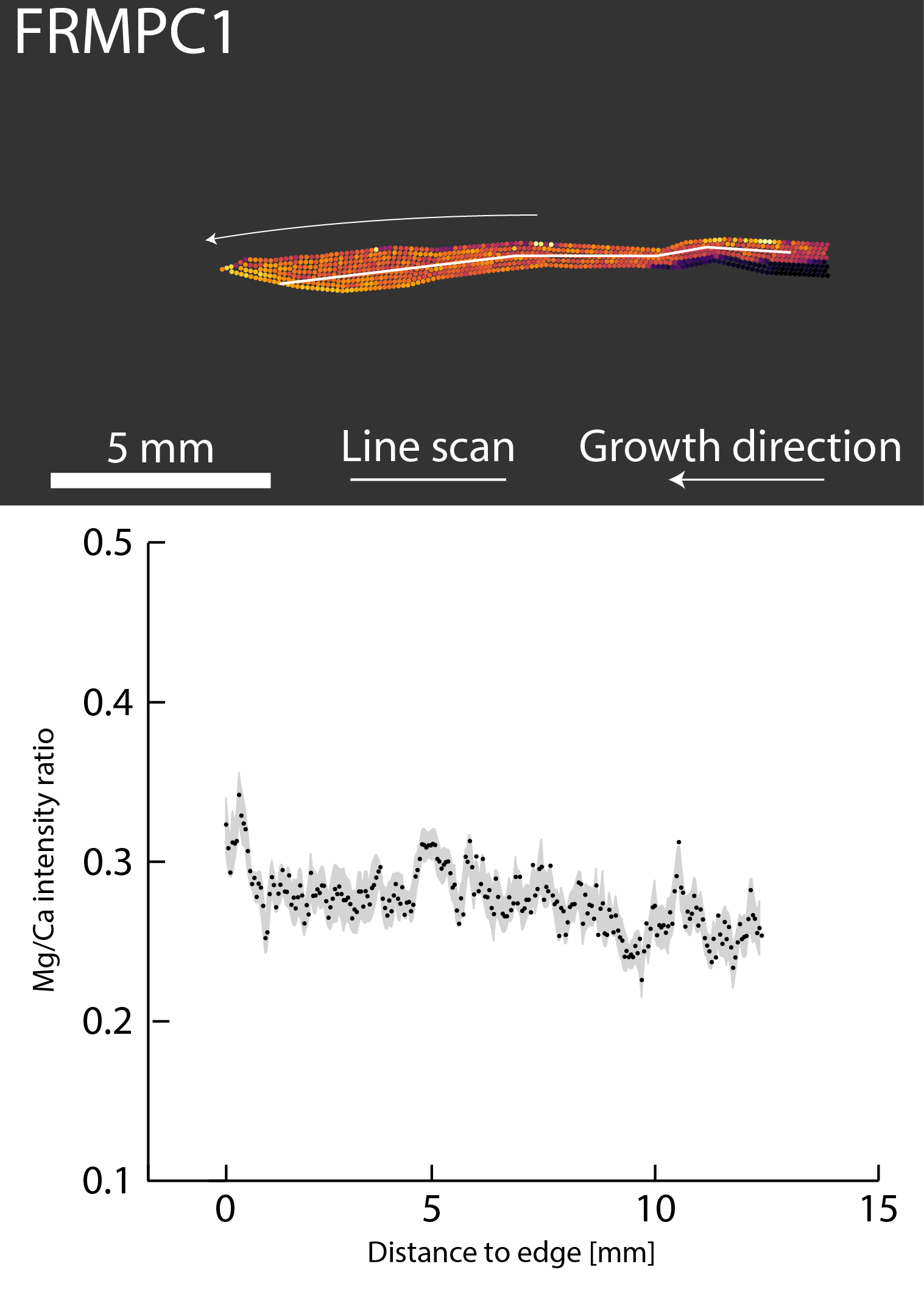


Supplementary Fig S3, Mg/Ca maps and line-scans following the direction of growth in partial sections of AKKPC3. Colours used for Mg/Ca intensity ratio range from lowest (black), to intermediate (purple) to highest (yellow) ratios found for each specimen. Line scans consist of average values from 5 spectra (black dot) and the standard deviation (grey) at each location. The line scan follows the centre of the M+2 layer from the growth edge towards the apex.


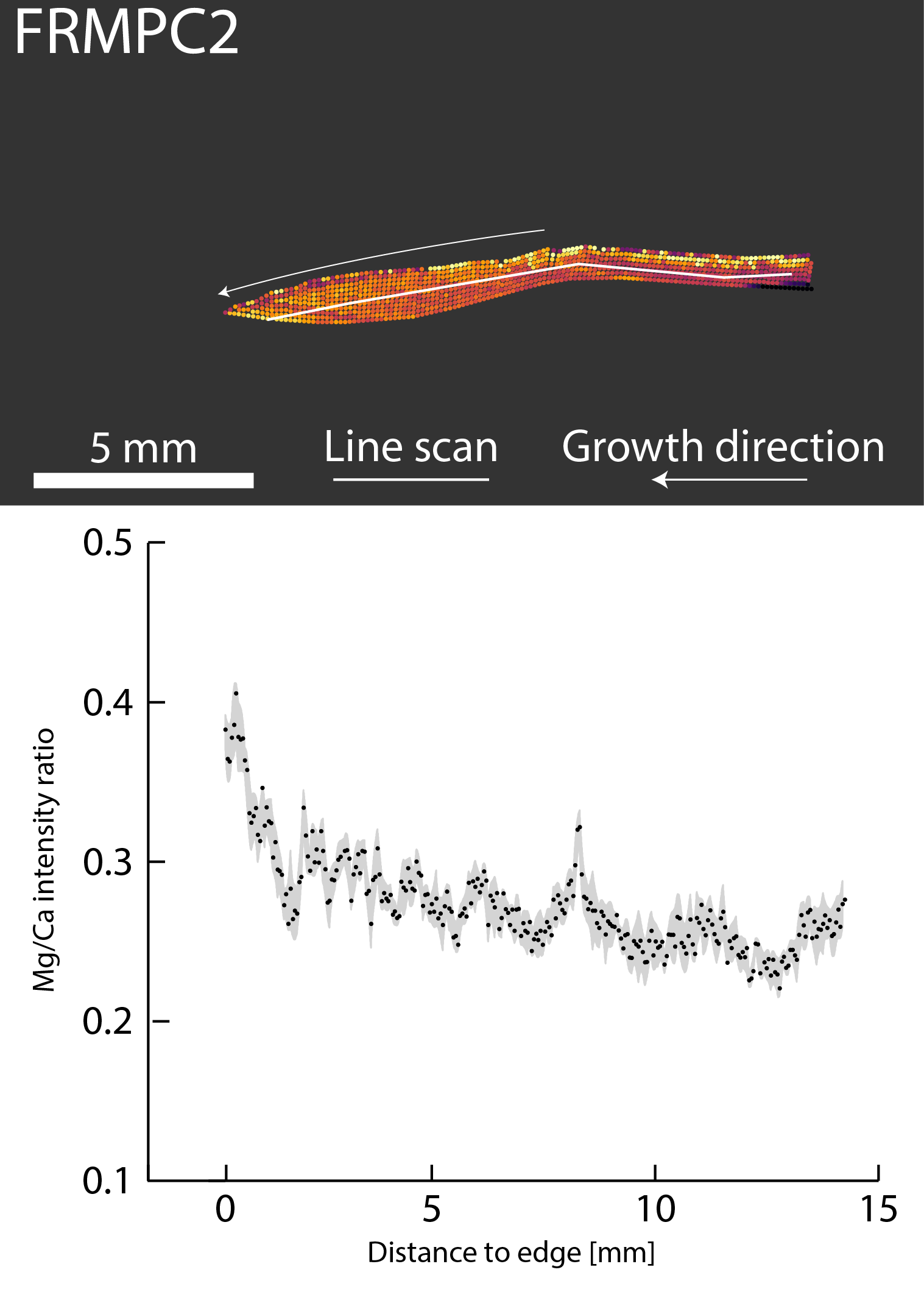


Supplementary Fig S4, Mg/Ca maps and line-scans following the direction of growth in partial sections of AKKPC3. Colours used for Mg/Ca intensity ratio range from lowest (black), to intermediate (purple) to highest (yellow) ratios found for each specimen. Line scans consist of average values from 5 spectra (black dot) and the standard deviation (grey) at each location. The line scan follows the centre of the M+2 layer from the growth edge towards the apex.


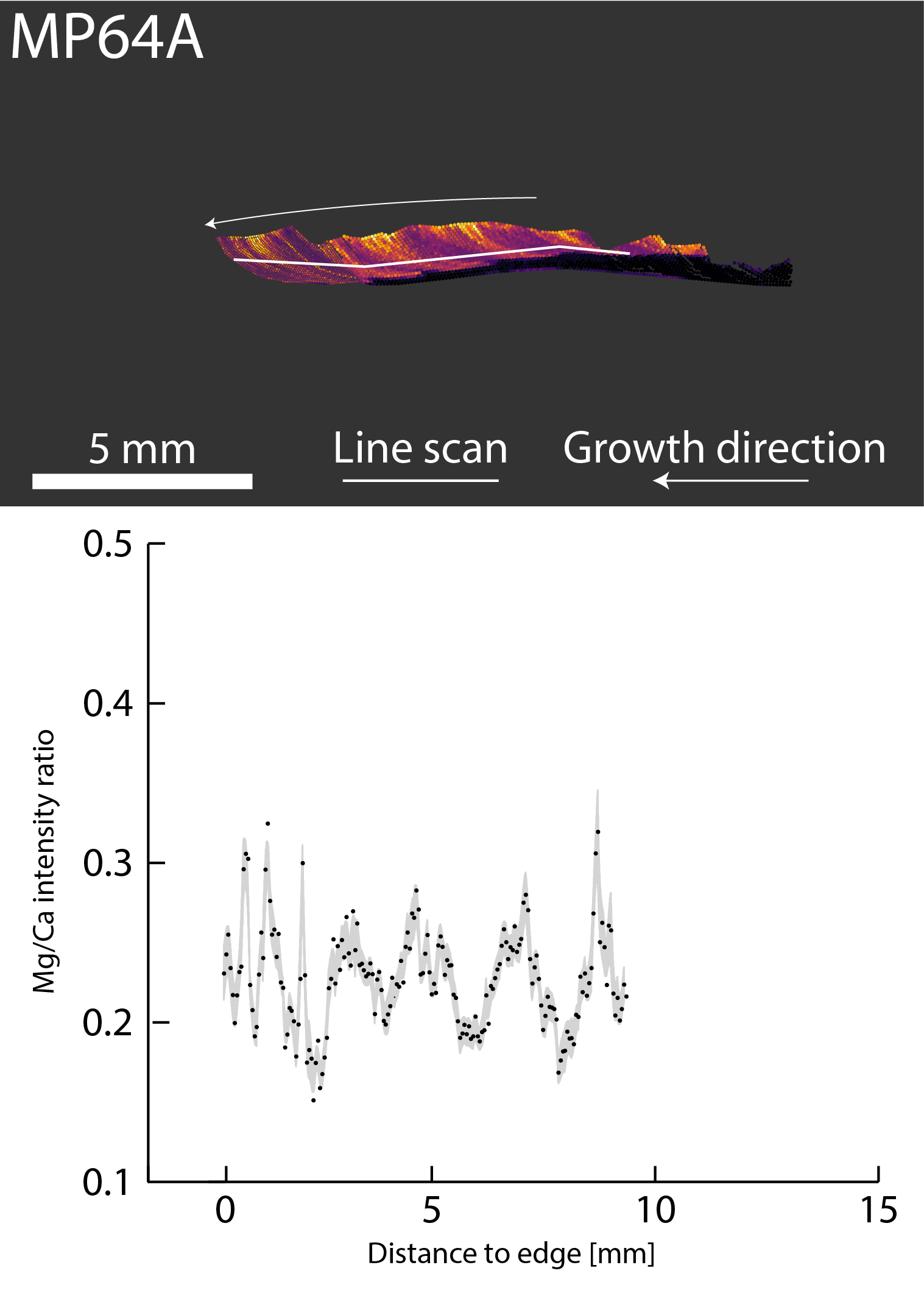


Supplementary Fig S5, Mg/Ca maps and line-scans following the direction of growth in partial sections of AKKPC3. Colours used for Mg/Ca intensity ratio range from lowest (black), to intermediate (purple) to highest (yellow) ratios found for each specimen. Line scans consist of average values from 5 spectra (black dot) and the standard deviation (grey) at each location. The line scan follows the centre of the M+2 layer from the growth edge towards the apex.


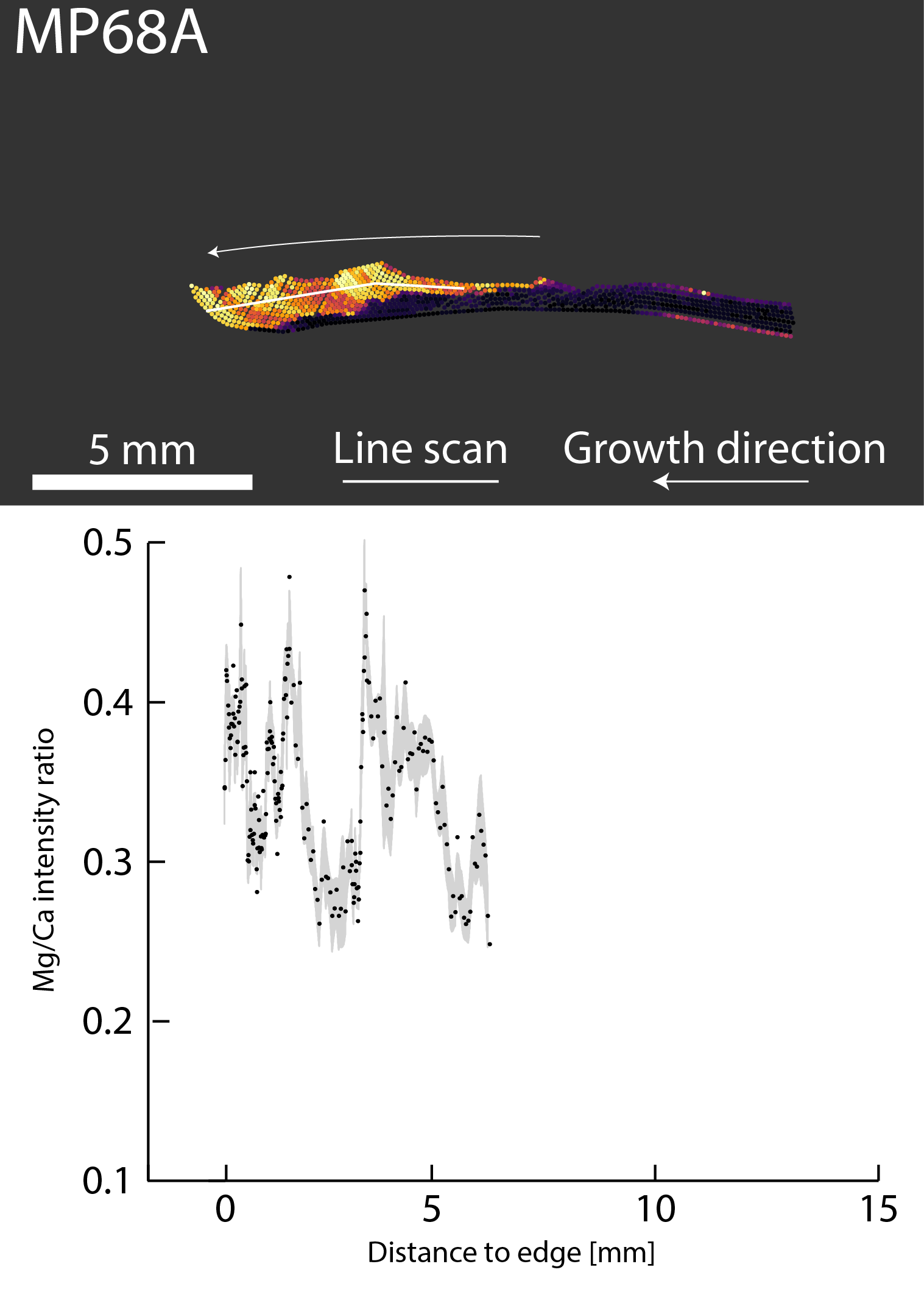


Supplementary Fig S6, Mg/Ca maps and line-scans following the direction of growth in partial sections of AKKPC3. Colours used for Mg/Ca intensity ratio range from lowest (black), to intermediate (purple) to highest (yellow) ratios found for each specimen. Line scans consist of average values from 5 spectra (black dot) and the standard deviation (grey) at each location. The line scan follows the centre of the M+2 layer from the growth edge towards the apex.


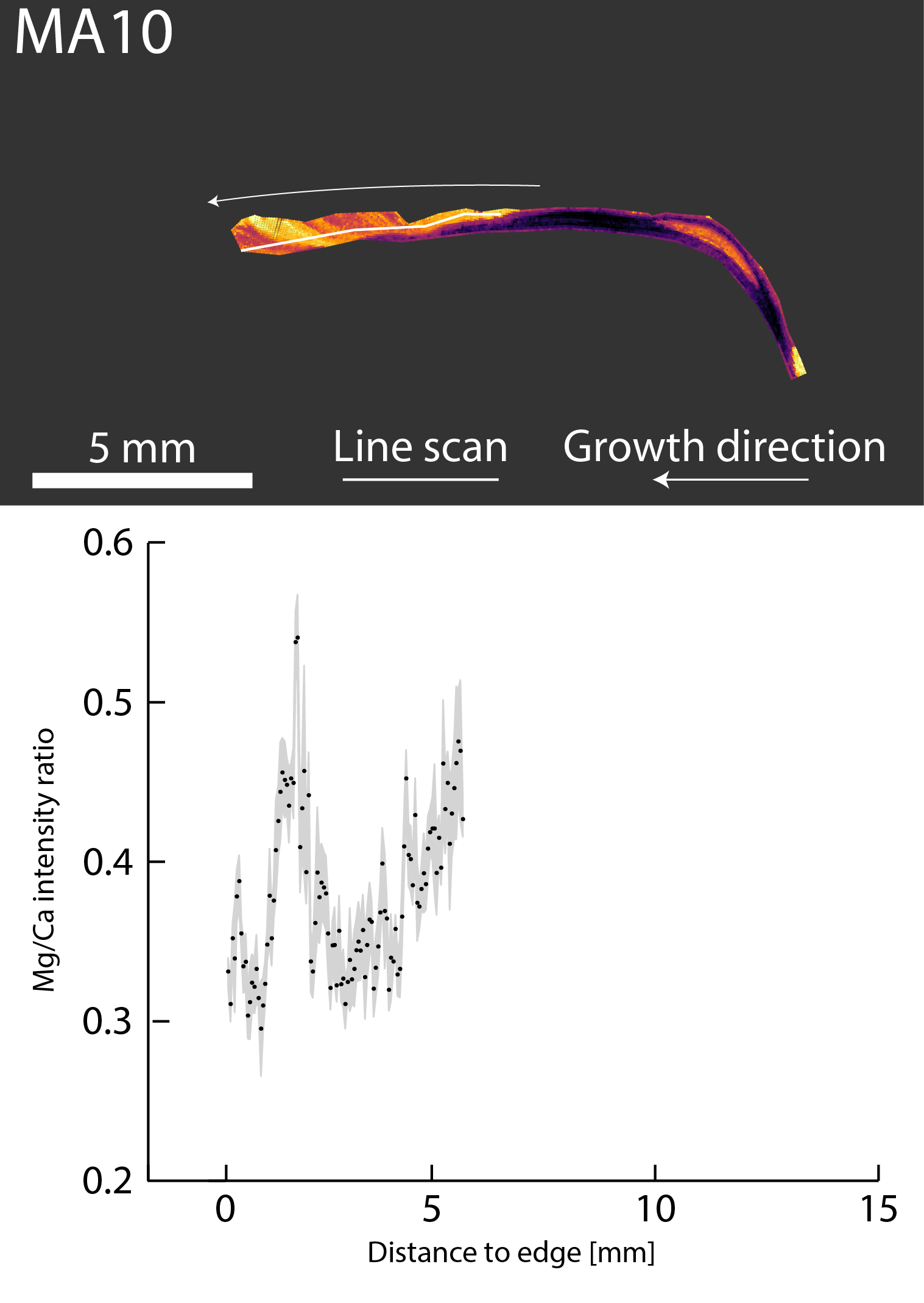


Supplementary Fig S7, Mg/Ca maps and line-scans following the direction of growth in partial sections of AKKPC3. Colours used for Mg/Ca intensity ratio range from lowest (black), to intermediate (purple) to highest (yellow) ratios found for each specimen. Line scans consist of average values from 5 spectra (black dot) and the standard deviation (grey) at each location. The line scan follows the centre of the M+2 layer from the growth edge towards the apex.


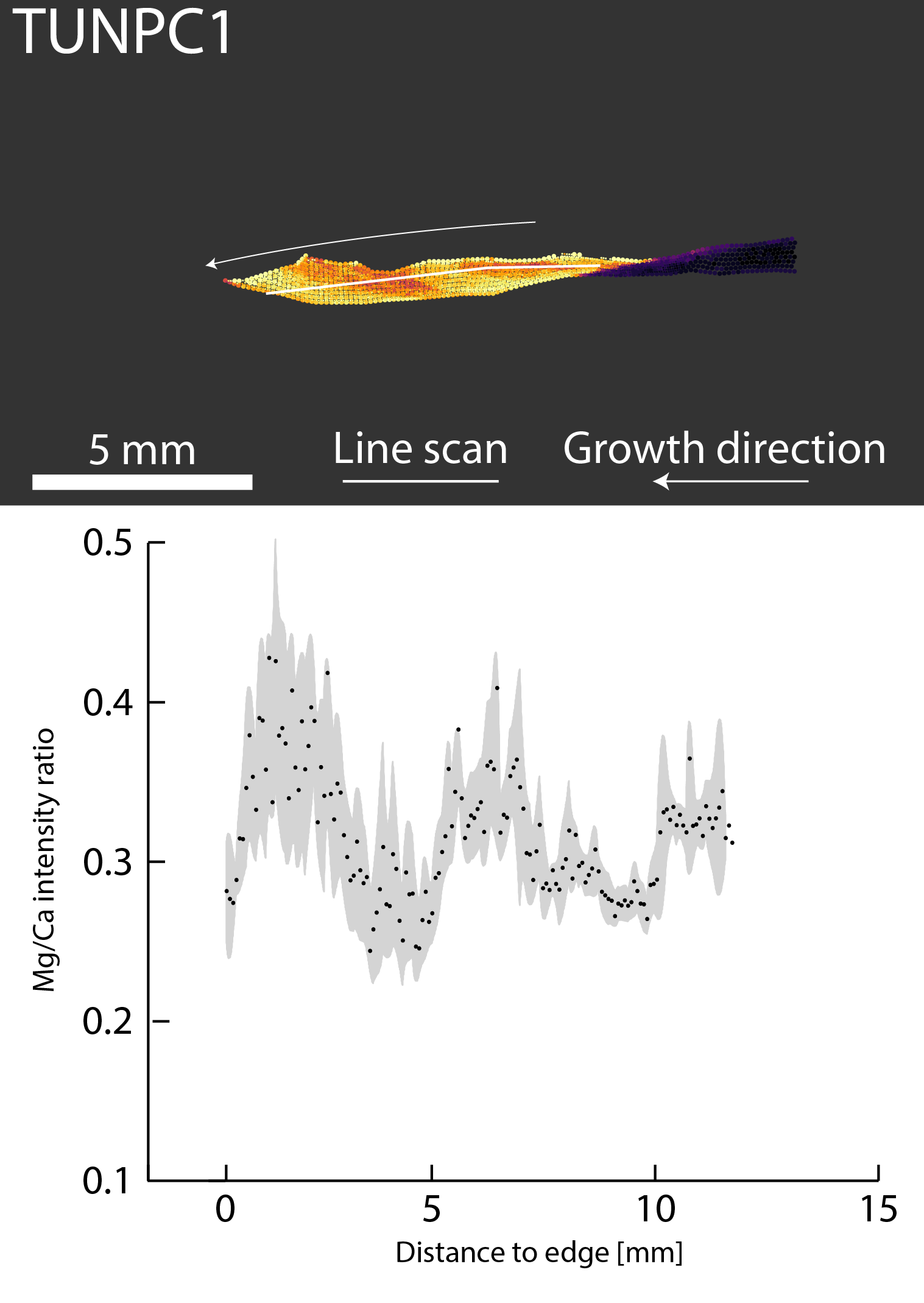


Supplementary Fig S8, Mg/Ca maps and line-scans following the direction of growth in partial sections of AKKPC3. Colours used for Mg/Ca intensity ratio range from lowest (black), to intermediate (purple) to highest (yellow) ratios found for each specimen. Line scans consist of average values from 5 spectra (black dot) and the standard deviation (grey) at each location. The line scan follows the centre of the M+2 layer from the growth edge towards the apex.


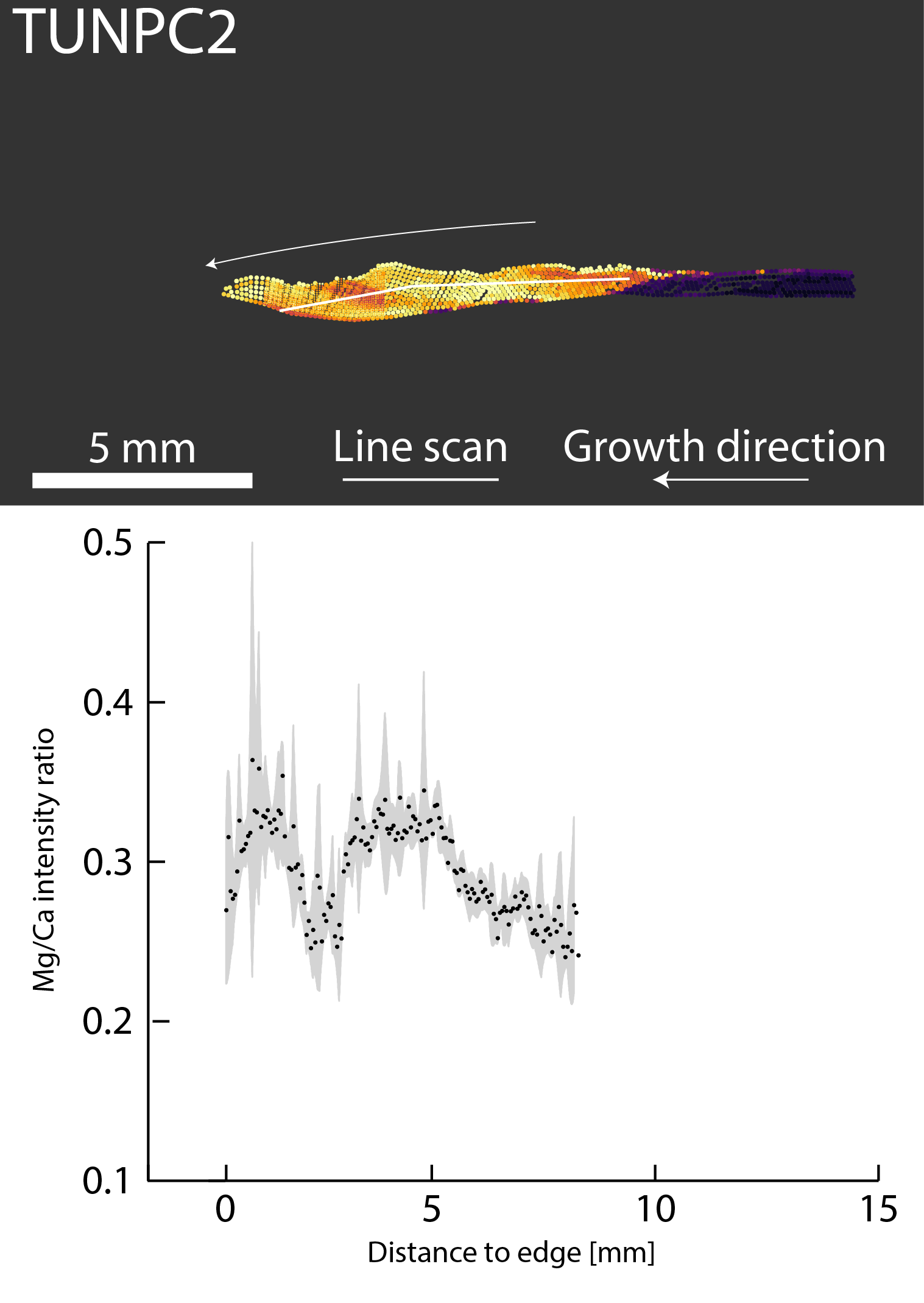


Supplementary Fig S9, Mg/Ca maps and line-scans following the direction of growth in partial sections of AKKPC3. Colours used for Mg/Ca intensity ratio range from lowest (black), to intermediate (purple) to highest (yellow) ratios found for each specimen. Line scans consist of average values from 5 spectra (black dot) and the standard deviation (grey) at each location. The line scan follows the centre of the M+2 layer from the growth edge towards the apex.


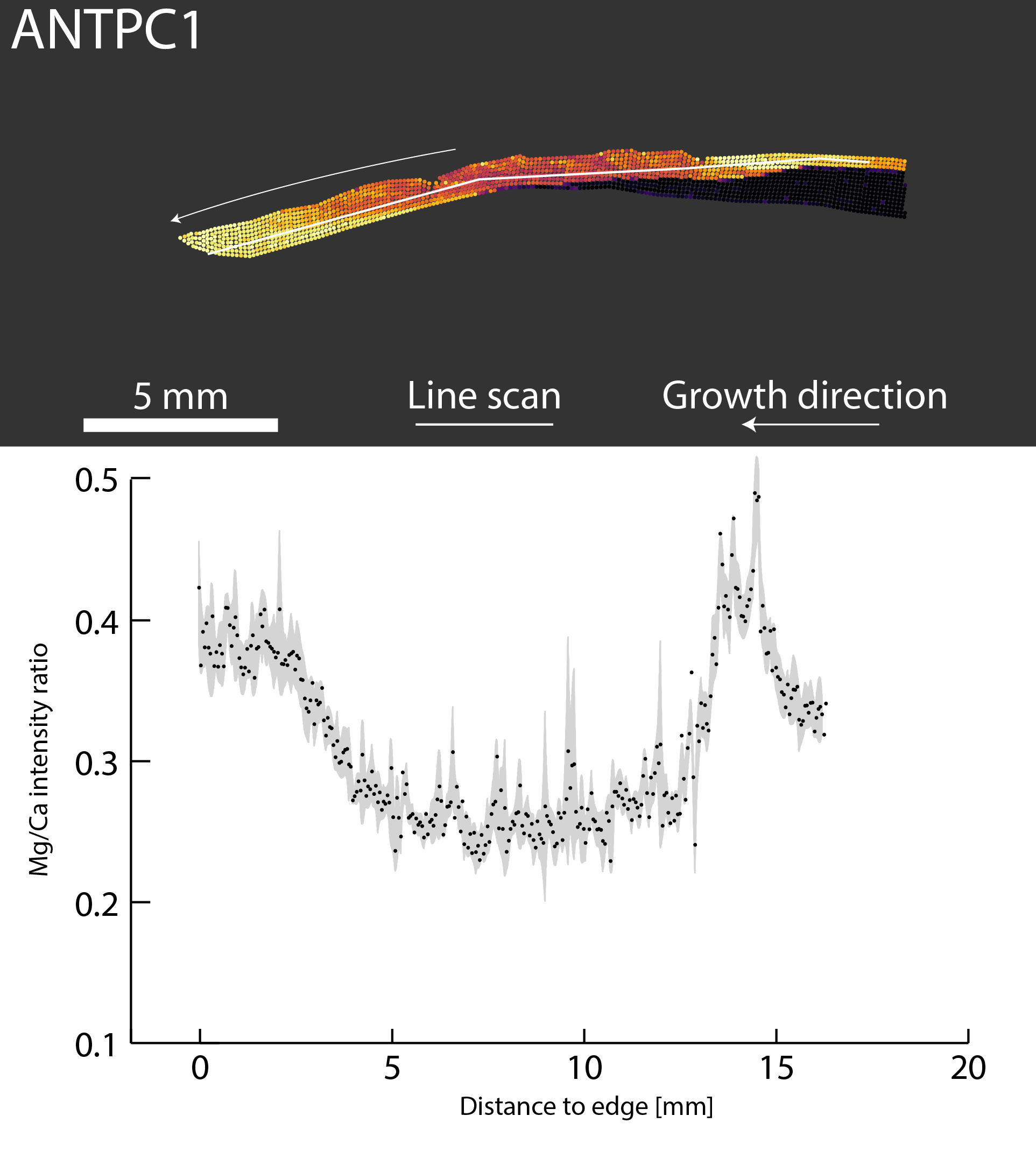


Supplementary Fig S10, Mg/Ca maps and line-scans following the direction of growth in partial sections of AKKPC3. Colours used for Mg/Ca intensity ratio range from lowest (black), to intermediate (purple) to highest (yellow) ratios found for each specimen. Line scans consist of average values from 5 spectra (black dot) and the standard deviation (grey) at each location. The line scan follows the centre of the M+2 layer from the growth edge towards the apex.


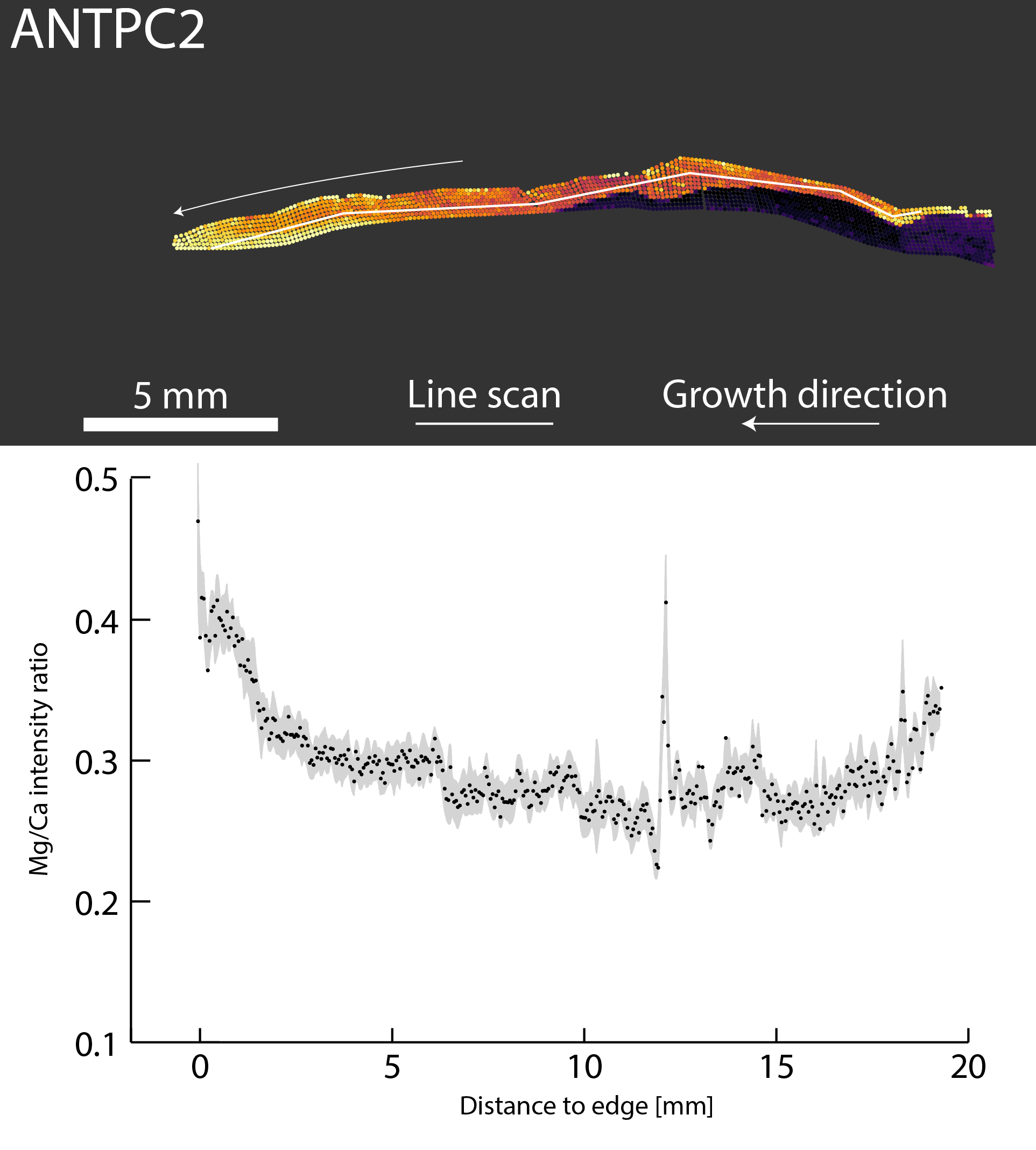


Supplementary Fig S11, Mg/Ca maps and line-scans following the direction of growth in partial sections of AKKPC3. Colours used for Mg/Ca intensity ratio range from lowest (black), to intermediate (purple) to highest (yellow) ratios found for each specimen. Line scans consist of average values from 5 spectra (black dot) and the standard deviation (grey) at each location. The line scan follows the centre of the M+2 layer from the growth edge towards the apex.


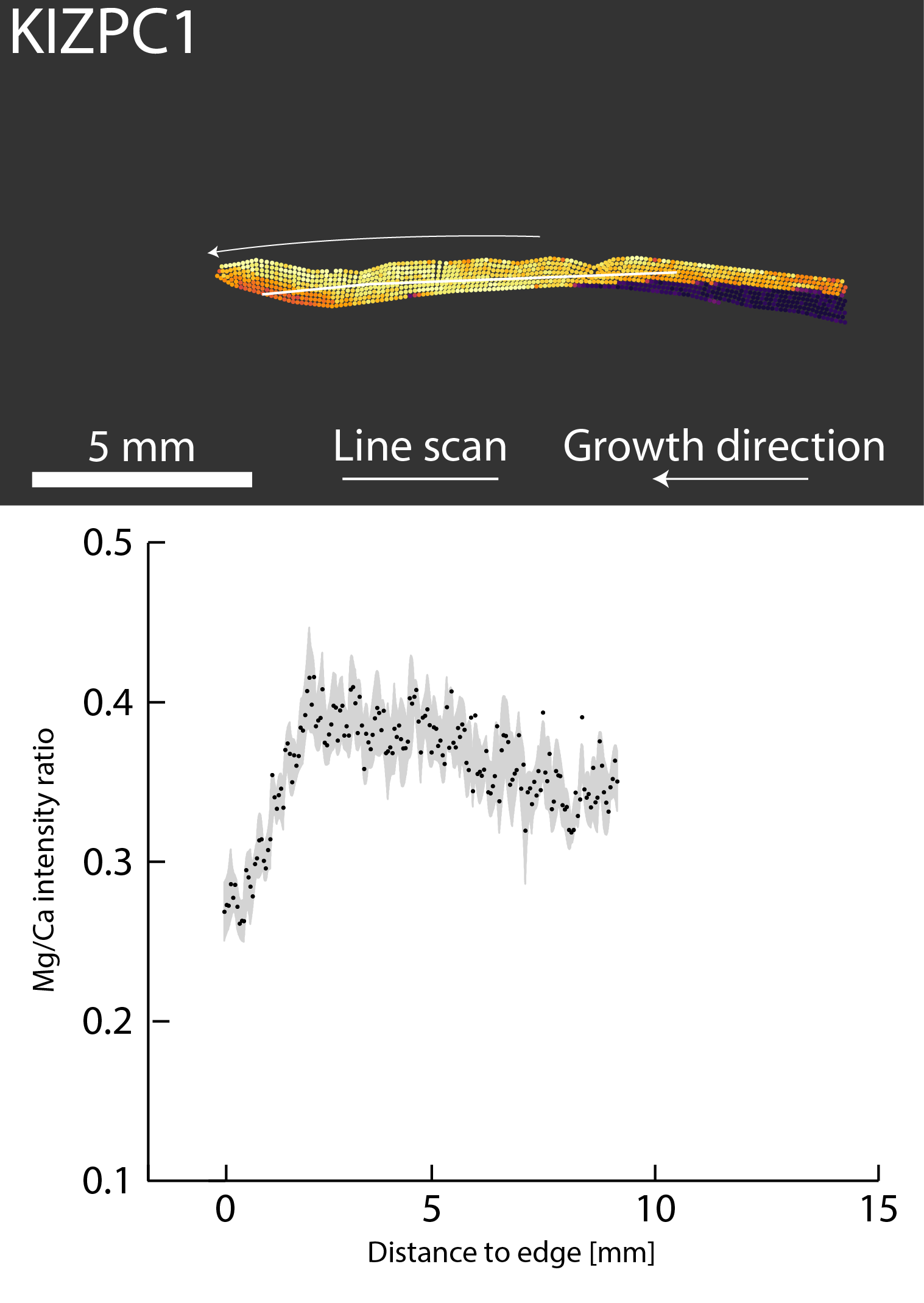


Supplementary Fig S12, Mg/Ca maps and line-scans following the direction of growth in partial sections of AKKPC3. Colours used for Mg/Ca intensity ratio range from lowest (black), to intermediate (purple) to highest (yellow) ratios found for each specimen. Line scans consist of average values from 5 spectra (black dot) and the standard deviation (grey) at each location. The line scan follows the centre of the M+2 layer from the growth edge towards the apex.


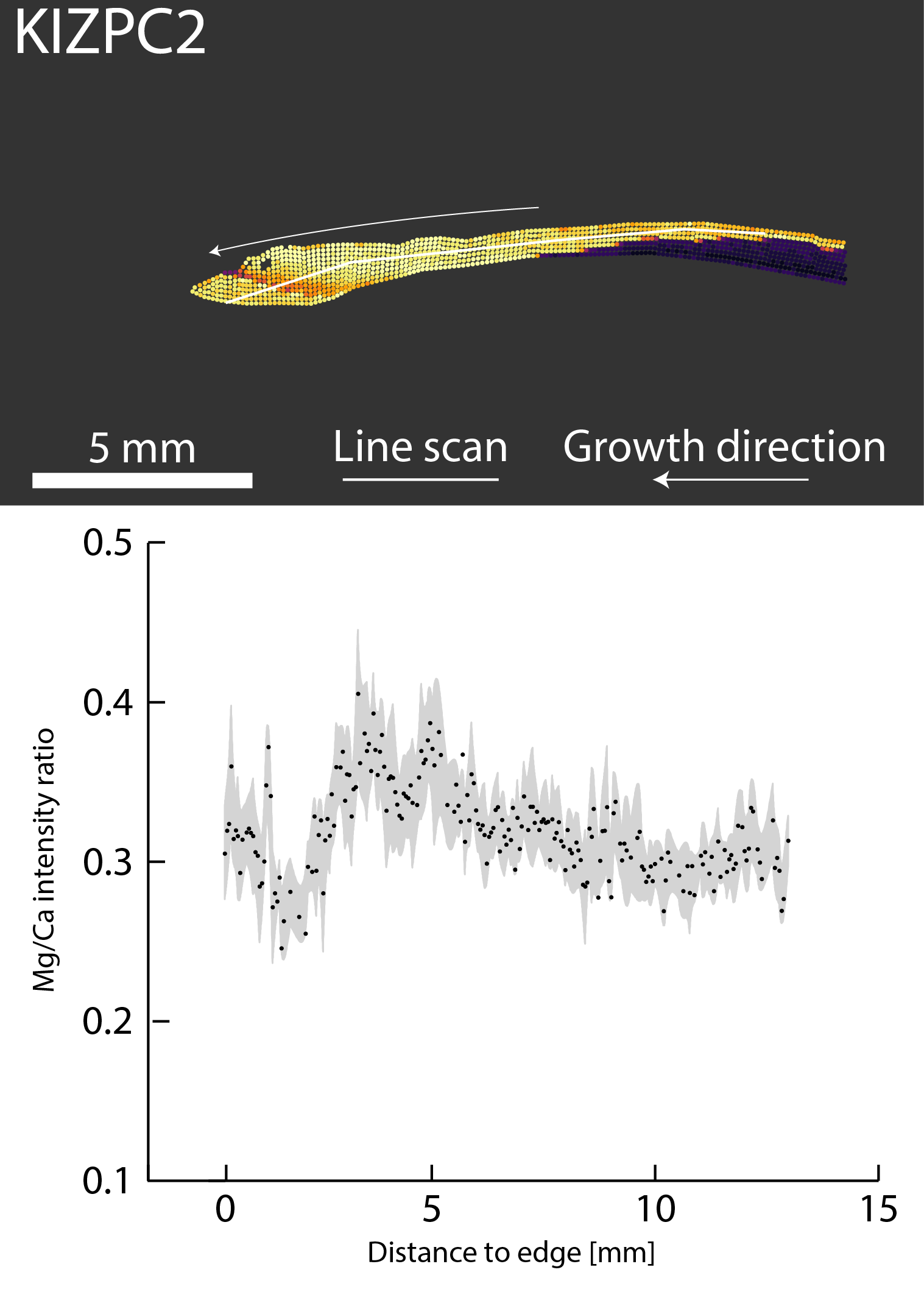


Supplementary Fig S13, Mg/Ca maps and line-scans following the direction of growth in partial sections of AKKPC3. Colours used for Mg/Ca intensity ratio range from lowest (black), to intermediate (purple) to highest (yellow) ratios found for each specimen. Line scans consist of average values from 5 spectra (black dot) and the standard deviation (grey) at each location. The line scan follows the centre of the M+2 layer from the growth edge towards the apex.


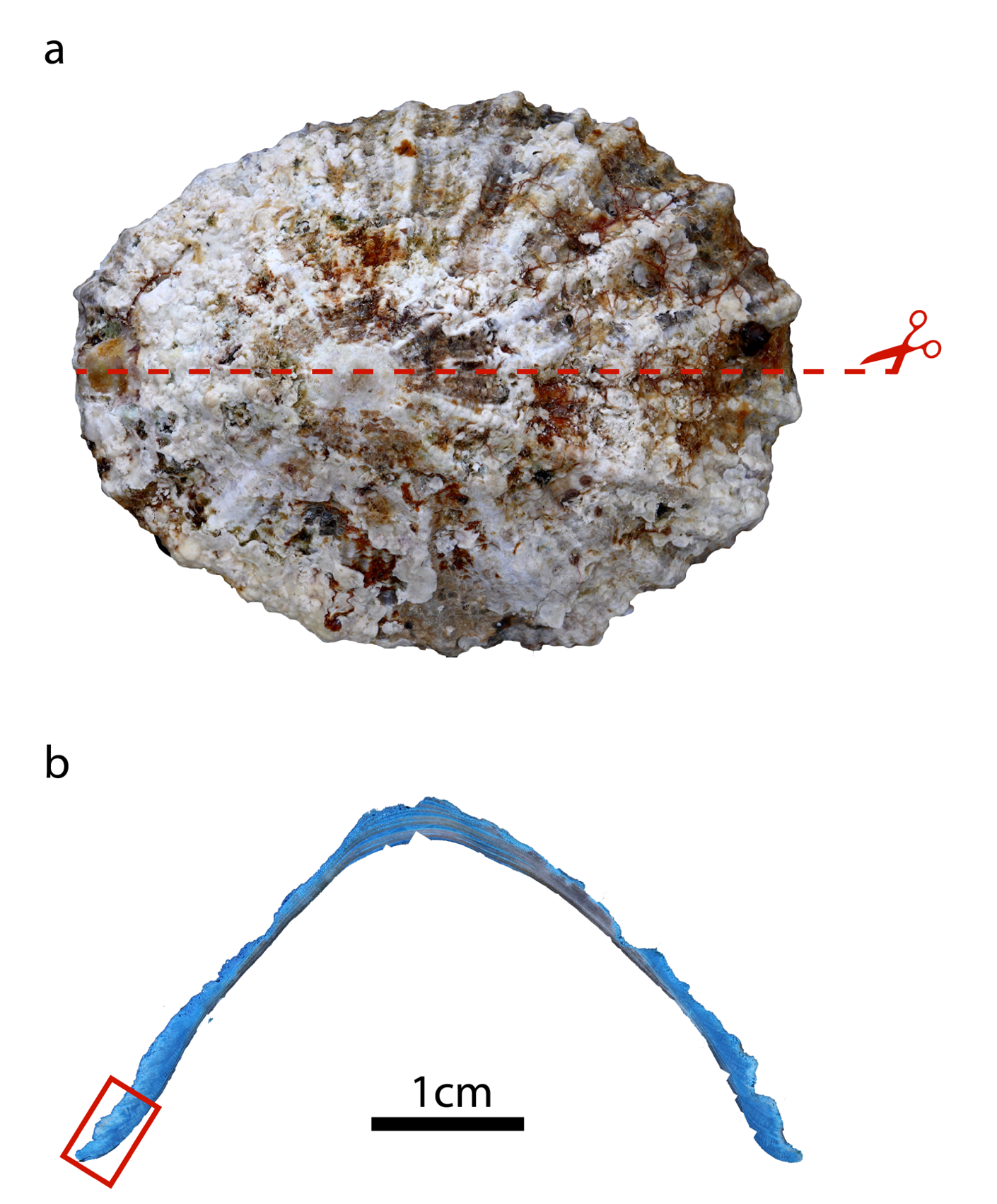


*Supplementary Fig S14, (a) location of exposed shell cross-sections on P. caerulea shell and (b) overview of cross-section after immersion in Mutvei's solution. Red rectangle indicates location of detailed imagein Fig S15c.*

*The image of the shell in a) was extracted from a photograph by H. Zell (license: CC-BY-SA-3.0* [*https://creativecommons.org/licenses/by-sa/3.0*](https://creativecommons.org/licenses/by-sa/3.0)*, and source:*[*https://upload.wikimedia.org/wikipedia/commons/1/15/Patella_caerulea_01.JPG*](https://upload.wikimedia.org/wikipedia/commons/1/15/Patella_caerulea_01.JPG) *)*


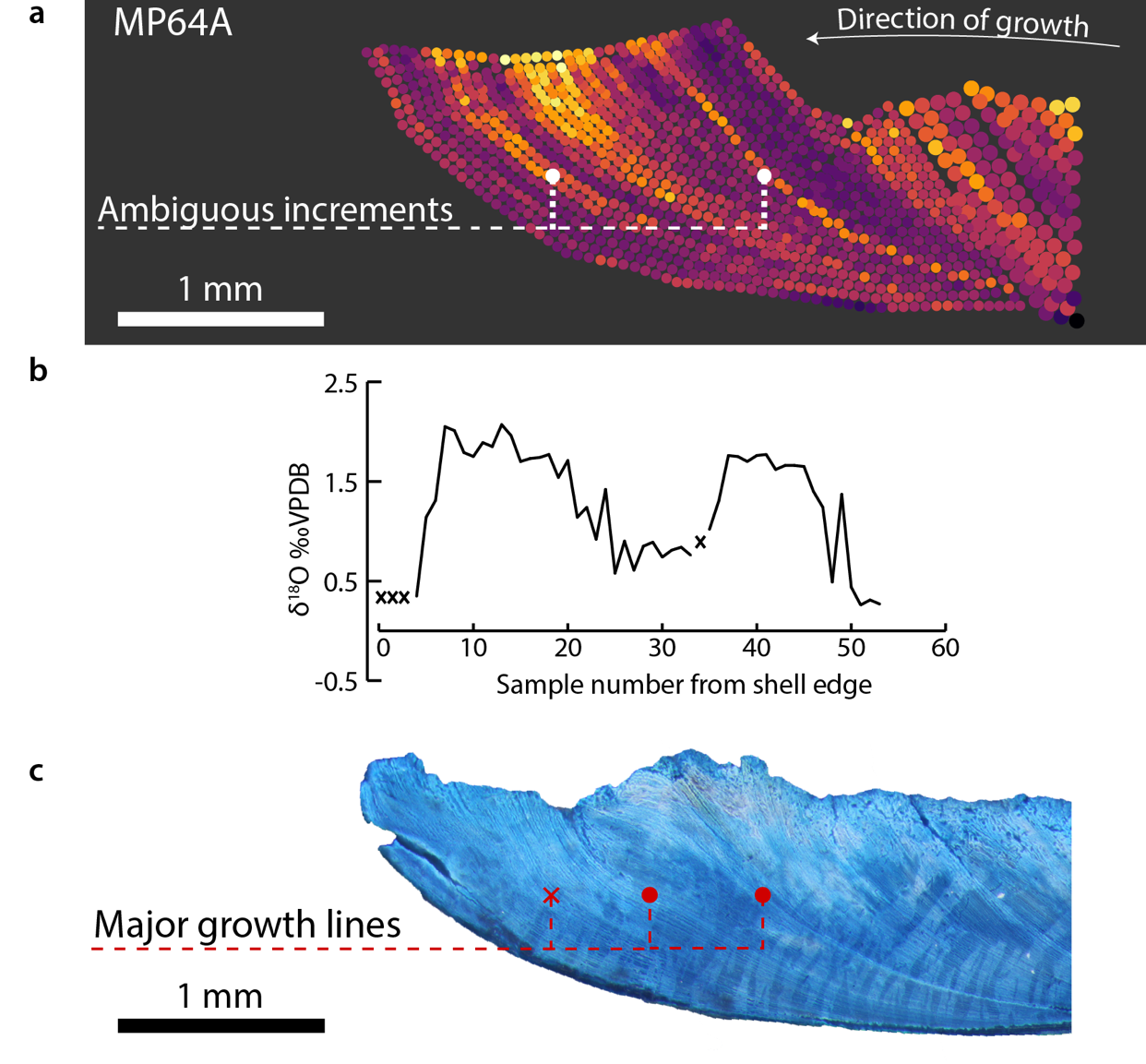


Supplementary Fig. S15, Comparison of increments with ambiguous enrichments of Mg/Ca (a) with oxygen isotope values (b) and stained section (c) from the same shell specimen. Note that while a,b and c are from the same specimen, they are from different parts that all reflect the last 2 years of growth. Specifically, a and b are from one half of the shell and from the posterior and anterior slope, respectively, while c is from the posterior slope of the other half of the shell, which was stained using Mutvei’s solution. Red circles indicate major growth lines, red cross indicates a lack of growth line.


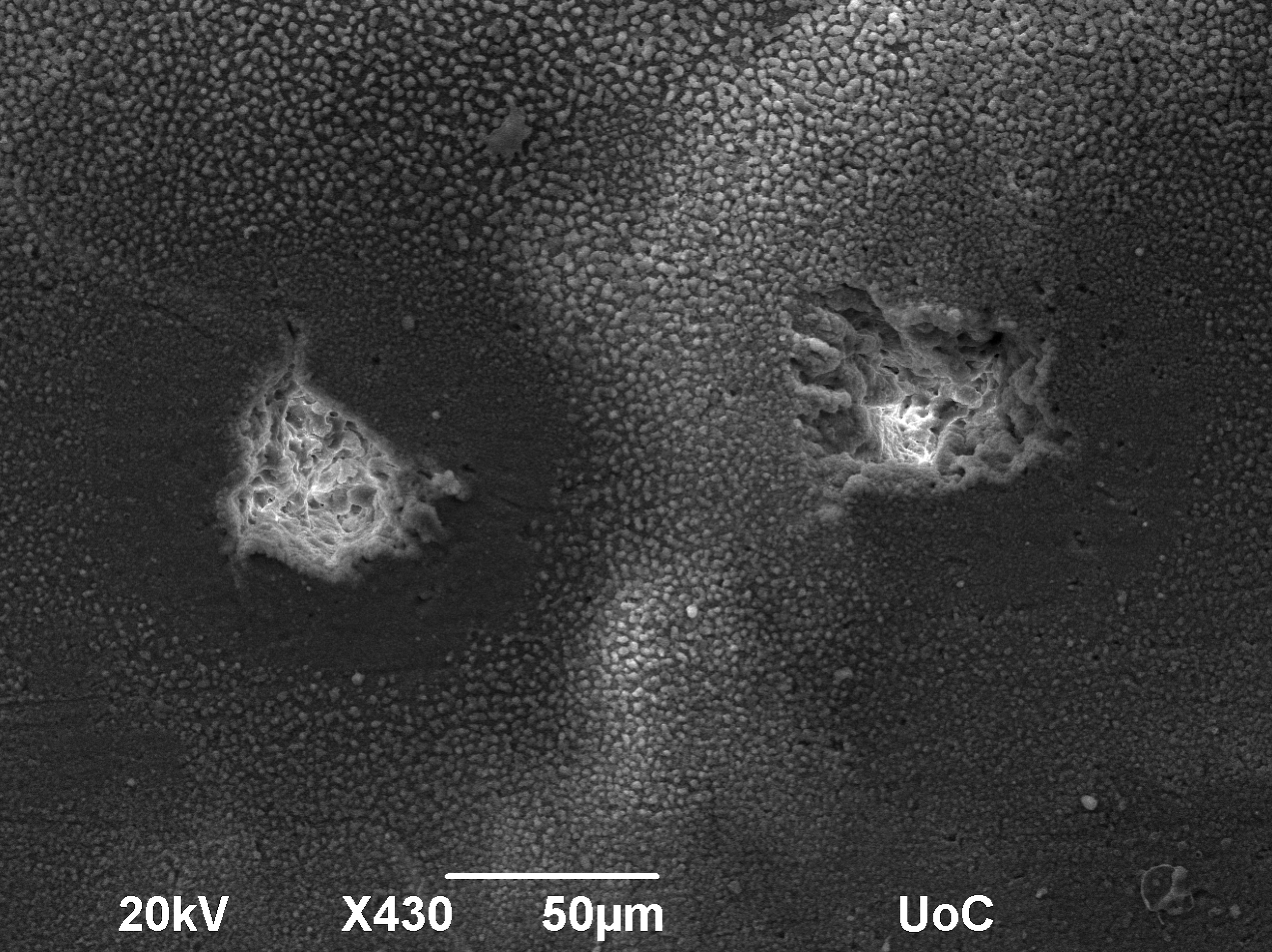


Supplementary Fig S16, Scanning electron microscope (SEM) image of laser ablation craters on the shell sample surface following irradiation with 5 laser pulses.

## Linear regressions between line scans for Mg/Ca intensity ratios and local SST measurements as well as between Mg/Ca intensity ratios and sequential δ^18^O values measured by Prendergast and Schöne^30^.

|  |  |
| --- | --- |
|  |  |
|  |  |
|  |  |
|  |  |
|  |  |
|  |  |
|  |  |
|  |  |
|  |  |
|  |  |
|  |  |
|  |  |
|  |  |
|  |  |
|  |  |
|  |  |
|  |  |
|  |  |
